# Did the elimination of lead from petrol reduce crime in the USA in the 1990s?

**DOI:** 10.12688/f1000research.2-156.v2

**Published:** 2013-10-08

**Authors:** Wayne Hall

**Affiliations:** 1 NHMRC Australia, The University of Queensland Centre for Clinical Research, Queensland, Australia

## Abstract

This article assesses the evidence for the hypothesis that a decline in all types of crime since the early 1990s in the USA was a consequence of removing lead from petrol between 1975 and 1985. It describes ecological and econometric studies that have generally but not always found correlations between lead exposures in childhood and some types of crime 20 years later; a small number of epidemiological studies that have found a dose-response relationship between lead exposure in childhood and self-reported and officially recorded criminal offences in young adulthood; and evidence for the biological plausibility of a causal relationship. Lead exposure in childhood may have played a small role in rising and falling crime rates in the USA but it is unlikely to account for the very high percentage of the decline suggested by the ecological studies. The major anomaly in the evidence is that the associations reported in ecological studies are much stronger (explaining 56-90% of the variation in crime rates) than the weaker relationships found in the cohort studies (that typically explain less than 1% of the variance in offending).  Suggestions are made for research that will better assess the contribution that reduced lead exposure has made to declining crime rates in the USA.

## Introduction

In the early 1990s the USA experienced a decline in crime that was unprecedented, and contrary to a predicted increase in crime ^1, 2^. Rates of all types of crime declined steeply, across all demographic groups and in all geographic regions in the USA ^2^. The decline may have been unexpected and unpredicted but, after the fact, there has been no shortage of possible explanations, the merits of which criminologists, economists and sociologists have debated (e.g. Blumstein and Wallman ^1^, Levitt ^2^, Drum ^3^, Nevin ^4^ and Reyes ^5^).

A number of factors have been accepted as contributing to the decline. These include: increased numbers of police and increased effectiveness of policing ^2^; the incapacitation of recidivist offenders who commit violent crimes by long prison sentences ^2, 6^; and a reduction in drug-related violence that followed the decline in crack cocaine use in major US cities in the late 1980s ^7, 8^.

A more controversial hypothesis was that the crime decline was a delayed demographic effect of more liberal abortion laws introduced in the USA in the early 1970s after the Roe vs Wade case in the Supreme Court ^2, 9^. Liberal abortion laws in the early 1970s, Donohue and Levitt ^9^ argued, reduced the number of unwanted male children who were born to unmarried mothers in US cities over subsequent decades. The absence of these children reduced crime rates 20 years later because there were fewer most-at-risk young men to commit violent offences.

Another explanation of the crime decline has recently been proposed ^2, 9^, which has one major advantage over other explanations, namely, that it claims to explain not only the steep decline in crime that began in the early 1990s but also the steep rise in crime that occurred between 1960 and 1990 ^3, 4, 10, 11^. According to this hypothesis, the rise in crime between 1960 and 1990 was driven by increased exposure of young children to environmental lead (primarily from leaded petrol and secondarily from leaded paint in old inner city housing) between the 1940s and the 1980s ^4, 11^. Crime increased 20 years later when the children exposed to lead entered young adulthood, the age at which the majority of crime is committed. The decline in crime after 1990 was an unexpected social benefit of removing lead from petrol in the USA over the period 1975 to 1985 ^5^.

## Why was reduced lead exposure a candidate explanation for the crime reduction?

The rationale for the hypothesis that lead exposure was criminogenic was that (1) lead exposure in childhood has been shown to reduce IQ and increase impulsiveness, aggressiveness and conduct problems in late childhood; and (2) these characteristics increase the risk that young people will commit criminal offences ^3, 5^.

The first observations of the neurological effects of childhood lead exposure were made in Brisbane in the early 1890s ^12^. For much of the 20 ^th^ century, lead-based paint was the most common source of lead poisoning in children ^13^. In the 1970s, epidemiologists found that elevated blood lead levels were associated with lower IQ and increased rates of behavioural disturbance ^14– 16^. Lead in petrol was then identified as the primary source of environmental lead exposure ^14, 16^.

The peak body of the lead industry contested these findings. The industry argued that: lead only caused harm at the high blood levels (80 g/dL) found in cases of lead poisoning; that lower lead levels did not cause harm because lead was a "natural" substance that the human body could efficiently excrete; that lead from paint was the major source of exposure in childhood, not lead from petrol; and that lead poisoning was a disease of poverty that occurred in the minority of children who ingested lead ^12, 14, 16^.

In 1975, the EPA specified that over a five year period, the average lead content of petrol would be reduced from 2 g to 0.5 g per gallon. In 1985, the EPA further reduced the allowable level, effectively removing all lead from petrol ^14^. The ban on the use of lead in petrol produced very steep declines in the amount of lead in petrol and blood lead levels in children between 1976 and 1980 ^15^. The fact that the steepest decline in blood lead levels coincided with elimination of leaded petrol made it hard for the lead industry to argue that leaded petrol was not a major source of environmental lead exposure in children.

## Evaluating the role of lead exposure in crime

In order to evaluate the hypothesis that lead exposure in childhood explains crime in young adulthood we need evidence that: (1) there is an association between lead exposure and crime; (2) the direction of the relationship is from lead exposure to crime rather than vice versa; and (3) enables us to exclude plausible alternative explanations of any association between childhood lead exposure and crime in young adulthood. If all of these conditions can be met, the case for a causal explanation is strengthened by the existence of a plausible mechanism by which lead exposure could increase crime.

### Evidence of an association between lead exposure and crime

***Ecological correlations.*** Nevin ^4^ reported ecological correlations between lead levels in the childhood environment, blood lead levels in children, and rates of crimes committed 23 years later. He showed that the time series curves for lead exposure and various types of crime (murders, rapes, aggravated assaults, and robberies) very closely followed each other when a 23 year lag was allowed between lead exposures in childhood and criminal activity in young adulthood. Regression analyses indicated that variations in childhood lead exposure explained large proportions of the variation in crime rates 23 years later (46% to 90%).

McCall and Land ^17^ tested Nevin’s ^4^ hypothesis by performing an age-period-cohort analysis that compared murder rates between 1960 and 1995 in 11 birth cohorts whose members differed in their lead exposure during childhood. They failed to find any relationship between lead exposure in childhood and murder rates, although the small number of birth cohorts that were available for analysis probably did not provide a powerful test of the hypothesis.

Nevin ^11^ extended his earlier analyses by analysing relationships between lead and crime in the USA and eight other developed countries (Australia, Canada, Finland, France, Italy, New Zealand, United Kingdom, West Germany) over the period 1960 to the early 1990s. He reported similar declines in crime rates in all these countries for all categories of crime (burglary and theft; aggravated assault, sexual assault, and murder). He identified an optimum lag between the elimination of lead in petrol and the onset of crime declines in all these countries as 19 years. The lag varied by type of offence, from 18 years for burglary to 23 years for robbery ^11^. Nevin also reported that lead levels during the 1970s predicted murder rates in large US cities in the period 1985–1994. The major weakness of Nevin’s analyses was his limited ability to control for the effects of other factors, apart from unemployment. He did have a crude control for national differences in crime rates between countries by including country as a dummy variable in his regressions.

***An econometric study of national US data.*** Reyes ^5^ attempted to address the confounding issue by conducting a detailed econometric analysis of relationships between lead exposure and violent crime over 50 US states during the period 1985–2002. She controlled for state differences in potential confounders and tested the robustness of her results to different ways of measuring lead exposure and different statistical models. Reyes’ estimated the lead content of petrol (g per gallon) in each state in each year and compared this measure with blood lead levels for the years in which these data were collected in the National Health and Nutrition Examination Survey (NHANES) (1976–1980) together with measures of ambient air lead within each state (1960–2000). Crime data were obtained from uniform crime statistics on property crimes, violent offences (assaults and robberies), and murders.

Reyes examined relationships between state crime levels and estimates of lead exposure in children in that state at lags of 20 and 30 years earlier. She controlled for differences between states in: rates of legalised abortion, unemployment, police numbers and incarceration rates, and also controlled for national trends in these factors over time (by including the study year as a variable in her model). She also tested the robustness of her findings to the choice of measure of lead exposure (namely, ambient lead vs lead in gasoline), the choice of statistical model (log-linear vs log-log), and the inclusion or exclusion of data from the states and territory whose large populations and high crime rates may have unduly affected the results (California, New York and Washington, DC).

Reyes found that the average levels of lead in gasoline and blood lead levels in children were correlated (r = 0.54) and the correlation increased to 0.84 if data from CA, NY and DC were excluded. She also found a close relationship, with a 20 year time lag, between the decline in lead exposure and the decline in violent crime. And she found that the largest declines in violent crime rates occurred in the states with the largest declines in lead exposure.

Unlike Nevin, Reyes did not find any relationship between lead levels and property crime. The relationship between lead levels and murder were also weak. There was no relationship between lead levels and murder rates when all 50 states were included in the analysis but there was a small effect when CA, NY and DC were excluded from the analysis. Reyes conjectured that this may reflect the contribution made to murder rates by gang-related drug violence in the major cities in these states and territories in the late 1980s.

Reyes estimated that the decline in lead exposure over the study period explained about 56% of the drop in violent crime between 1992 and 2002. She also estimated that legalized abortion accounted for 29% of the drop and that increased police numbers and decreased alcohol use accounted for a smaller proportion of the decline.

Reyes concluded that lead exposure in childhood was causally related to rates of violent crime (assaults and robberies) committed 20 years later when children exposed to lead in childhood entered the peak period of criminal offending. She concluded that "lead exposure was likely an important factor in both the rise and the decline of violent crime in the last 30 years" and that "two major acts of government, the Clean Air Act and Roe v Wade, neither intended to have any effect on crime, may have been the largest factors affecting violent crime trends at the turn of the century" (p 36).

Reyes’ model of the effects of lead exposure on crime over-predicted the observed crime decline, that is, according to her model, crime rates should have declined by 56% as a result of the decline in environmental lead but the observed rate of decline was only 34%. She hypothesized that there were other countervailing factors at work that increased crime rates at the same time that declining lead exposure was driving them down.

***Ecological studies of large cities.*** The ecological analyses of Nevin and Reyes suffer from the limitation that they use a single estimated lead exposure for the whole of a state. This ignores the fact that lead exposures differ greatly between the populations of large cities, small cities and rural areas within states. Mielke and Zahran ^18^ attempted to address this problem by analysing relationships between measures of ambient lead and aggravated assaults per 100,000 people in six large US cities (Atlanta, Chicago, Indianapolis, Minneapolis, New Orleans and San Diego) ^18^. They argued that aggravated assault was more likely to be impulsive and hence reflect the effects of childhood lead exposure. They estimated annual city burdens of air lead from data on vehicle traffic in metric tons, state gasoline usage, city traffic volume, average miles per gallon and lead per gallon of different types of fuel. They followed Reyes in forward lagging effects of lead exposure on crime rates by 22 years and they controlled for income per capita, and the percentage of city population age group most at risk of committing these offences (15–24 years).

Mielke and Zahran found that ambient lead levels in these cities predicted aggravated assault rates, after controlling for income and demography and baseline differences in crime rates between cities. They estimated that between 66% and 89% of the variations in aggravated assault rates in these cities were explained by variations in ambient lead levels. They also estimated (making worst case assumptions about the contribution of lead paint to ambient lead) that leaded petrol accounted for at least 85% of the lead to which children were exposed in New Orleans, the city with highest ambient lead.

### Epidemiological studies of lead and crime

Sceptical researchers (e.g. Fergusson ^19^ and Firestone ^20^) have argued that it is hazardous to draw causal inferences about lead exposure and crime from correlations between time series data on lead exposures and crime rates. To do so is to run the risk of committing the ecological fallacy, that is, mistakenly assuming that a correlation between two time series necessarily means that the people committing the crimes are those who were exposed to lead in childhood.

Epidemiological studies rule out the ecological fallacy by examining the relationship between lead exposure and rates of crime in individuals. They thereby enable us to see if it is the individuals with highest lead exposure who commit the most crimes. They also can address the contributions of potential confounders to the relationship by assessing whether lead exposure in childhood predicts crime rates in young adulthood, after controlling for other risk factors for lead exposure and crime.

Needleman *et al.* ^13^ reported a case-control study on lead exposure and crime. They compared lead levels in 194 youths aged 12–18 who were "adjudicated" delinquents (i.e. had been found guilty of criminal offences) and 146 non-delinquent control subjects from high schools in the same city. They also measured factors that predict delinquency such as: race, parental education and occupation, number of parents in the home, number of children in the home, and neighbourhood crime rates. They found a large difference in lead concentrations between delinquents and controls in both black and white participants (11 vs 1.5 parts per million (ppm)). The delinquent participants were 1.9 times more likely to have a lead level above 25 ppm than controls. Delinquency was confounded by race: fewer black than white controls were recruited, and more black delinquents came from single parent families and lived in higher crime areas than white delinquents. When potential confounders were included in the model, the strength of the relationship between lead level and delinquency *increased* to an odds ratio of 4.0 (95% CI: 1.4, 11.1) and lead level was the second strongest predictor of delinquency after race. Lead was the second strongest predictor of delinquency (after being raised by a single parent) when separate analyses were carried out within the black and white samples.

Wright *et al.* ^21^ conducted a prospective study of the relationship between lead exposure before birth and until age 6.5 years and records of arrest in 250 primarily African American individuals (90%) who were followed from age 19 to 24 years. Participants were in a cohort study of children born to 376 pregnant women recruited from four prenatal clinics in socially disadvantaged areas of Cincinnati, Ohio. In an earlier follow up of 195 members of the cohort, Dietrich *et al.* ^22^ reported a dose-response association between measures of childhood lead exposure and acts of delinquency from self-report and parental report by age 18 years. These relationships persisted after adjustment for confounders.

Wright *et al.* examined delinquent acts that resulted in arrests in the county in which the participants lived. They found elevated lead levels (13.4 µg/dL on average during childhood) and an elevated risk of arrest in their sample (54% had been arrested a total of 800 times, 14% for violent offences). The risk of arrest increased with blood lead, after adjustment for: sex; quality of home environment; maternal alcohol, tobacco and illicit drug use during pregnancy; maternal arrests rates, socioeconomic status (SES), number of children in the home and whether the mother was on welfare. The risk of any arrest increased 1.40 times for each increase of 5 µg/dL in prenatal blood and 1.27 for each increment in average blood lead level during childhood. The risk of arrest for violent offences increased 1.34 times for each 5 µg/dL increase in antenatal blood lead, and 1.48 for each 5 µg/dL increase in average blood lead during childhood.

Fergusson *et al.* reported a prospective study of the relationship between childhood lead exposure between 6 and 9 years, measured in deciduous teeth, and (i) convictions and (ii) self-reported criminal activity between the ages of 14 and 21 years in a New Zealand birth cohort of 1011 individuals. They found dose-response relationships between lead in teeth and both convictions for property and/or violent offences and self-reported rates of property and/or violent crimes. The relationship was stronger for self-reported crime, probably because conviction rates were much lower. Relationships persisted after adjustment for confounders (maternal education, ethnicity, family conflict, maternal smoking during pregnancy, exposure to childhood physical abuse, parental alcohol and drug use and offending).

Fergusson *et al.* also tested a plausible causal pathway for the effects of lead on crime. They assessed whether the relationship between lead dentine levels and crime could be explained by a decline in IQ produced by lead exposure. Adjustment for educational failure eliminated the association between self-reported crime and lead levels but the association with convictions persisted, albeit much attenuated. They estimated lead exposure explained less than 1% of the variance in convictions and self-reported crime and argued that this was too weak to explain Nevin’s very high estimates of the percentage variation in crime rates attributed to lead exposure (63–90%) ^11^.

### Excluding plausible alternative explanations

Lead exposure and violent crime are correlated in ecological studies and in a small number of epidemiological studies in the USA and New Zealand. The prospective epidemiological studies indicate that lead exposure in childhood precedes criminal acts in young adulthood but it is much more difficult to exclude other plausible explanations of the association. Foremost among these is the possibility that the association can be explained by uncontrolled factors that increase the likelihood of being exposed to lead and of committing crimes (e.g. living in disadvantaged high crime inner city areas where lead exposure is common). This is a special challenge in the USA because the most socially disadvantaged African-American populations live in inner city areas with high levels of exposure to dust from lead-pigmented paints and, until the late 1970s, high levels of lead from leaded petrol from the freeways that ran through inner city housing in large US cities ^11^.

Nevin’s ecological studies had limited ability to address this confounding. Reyes controlled for some confounding factors using state data on unemployment, number of police officers and so on. Potential confounders were better controlled in two of the prospective epidemiological studies. In the Wright *et al.* study, the whole sample came from a very socially disadvantaged population and had high average lead exposure throughout childhood. Yet Wright *et al.* found a relationship between lead exposure during childhood and arrest for all crimes, and for violent crimes in adolescence and young adulthood. These relationships persisted after controlling for potential confounders.

The Fergusson *et al.* study was conducted in a very different cultural setting. Christchurch in New Zealand has much lower rates of social disadvantage than Cincinnati in the USA, a good social welfare safety net, and a very different minority ethnic group with a higher rate of criminal convictions. There were also much lower average lead levels in Christchurch than in Cincinnati but Fergusson *et al.* nonetheless found a dose-response relationship between lead exposure in childhood and rates of criminal convictions and self-reported criminal offences committed by age 21. These relationships persisted after adjustment for confounders. There was also a plausible mechanism in that controlling for IQ reduced the strength of the relationship between lead and rates of self-reported criminal offences.

### Biological plausibility

There is a consilience of evidence in support of the hypothesis that lead exposure in childhood increases the risk of criminal offending in young adulthood. Low intelligence increases the risk of offending, and there is a dose-response relationship between lead exposure in childhood and a decline in average IQ ^13, 19, 23^. Fergusson *et al.* ^19^ also showed that the relationship between dentine lead levels and self-reported crime was no longer significant after adjusting for school completion.

There is also support for a second way that lead exposure may increase offending, namely, that lead exposure in childhood makes adolescents more impulsive, hyperactive and aggressive. Meta-analyses find small but consistent correlations between blood and dentine measures of lead exposure and symptoms of conduct problems (r = 0.15) ^24^, inattentiveness (r = 0.14) and hyperactivity (r = 0.12) ^25^. These patterns of behaviour in turn increase the risk of antisocial behaviour in adolescence and young adulthood ^26^. A recent neuroimaging study of the Cincinnati cohort reported correlations between childhood lead exposure and the size of frontal areas in the brain implicated in executive functioning, mood regulation and decision-making ^27^.

## Does the decline in lead exposure explain the decline in crime in the USA?

Eliminating lead from petrol in the USA between 1975 and 1985 dramatically reduced lead exposure in childhood and this occurred over a period that could explain the post-1990 decline in crime in the USA. Ecological studies have generally but not always found correlations between lead exposures in childhood and crime rates 20 years later. But these studies have not found the same relationships between lead exposure and crime. Nevin ^11^ found a relationship for *all* types of crime (violence, sexual assault, murder and property crime) but Reyes only found a relationship for violent offences and there was only a suggestive relationship for murder. Mielke and Zahran’s supported Reyes findings in their analysis of aggravated assault in six US cities. McCall and Land’s ^17^ age-period-cohort analysis found no association between lead exposure and rates of murder in their age-period-cohort analysis.

There are a small number of epidemiological studies that find dose-response relationships between lead exposure in childhood and both self-reported and officially recorded criminal offences in young adulthood. A causal relationship is biologically plausible because lead is neurotoxic in animals, produces hyperactivity, and impairs learning ^28^. There is now suggestive human neuroimaging evidence that lead exposure is related to size reductions in brain regions involved in executive functioning and decision making ^27^.

But the evidence is not sufficient to conclude that variations in environmental lead exposure in childhood over the past 50 or so years in the USA explain, first the rise, and then the decline in crime rates. The major reason for doubt is that the associations in ecological studies are much stronger (explaining 60–90% of the variation in crime rates) than the weaker relationships in the cohort studies (that explain less than 1% of the variance in offending) ^19^. Lead exposure in childhood may have played a small role in rising and falling crime rates in the USA but it is unlikely to account for the very high percentage of the decline suggested by Nevin ^11^ and Reyes ^5^.

This issue is not solely a matter of interest only to criminologists. Understanding the reasons for the US crime decline has important implications for social policy. If lead exposure is a major cause of the decline, then we could avoid mistaken investments in crime reduction strategies that have been erroneously given the credit for the crime decline. We would also need to weigh the costs and benefits of different ways of reducing residual childhood lead exposures in inner city areas.

The contribution of lead exposure to crime rates deserves serious research attention. We need more cohort studies in environments where lead exposure remains high, particularly in developing countries such as China. The results of these studies could inform epidemiological modelling to estimate the likely population level effects on crime of observed reductions in lead exposure. This modelling would test the plausibility of the lead hypothesis. So too would controlled evaluations of the costs and benefits of reducing environmental lead exposure in high crime inner city areas or moving highly exposed populations from areas of high lead burden.
