# Supplementary material for: Did the elimination of lead from petrol reduce crime in the USA in the 1990s?
Source: F1000Res. 2013 Oct 8;2:156. Originally published 2013 Jul 16. [Version 2] doi: 10.12688/f1000research.2-156.v2 (PMC3829390; doi:10.12688/f1000research.2-156.v2)
Supplement: Peer review history

# Peer review: Referee response for version 2

**DOI:** 10.5256/f1000research.2664.r2718

**Published:** 2013-12-10

**Authors:** Kenneth Land

**Affiliations:** 1 Population Research Institute, Duke University, NC, USA

The revision to this article clarifies its conclusion. My commentary on the previous version to the effect that both criminology and epidemiology perspectives are important for an understanding of crime trends in the U.S. (and the extent to which the presence of lead in petrol was a contributing factor) still holds. 

For instance, consider the data plotted in Figure 1: this rate is calculated annually per 1,000 individuals in the 12 to 17 age group and is based on the perceived ages of the offenders as estimated by violent crime victims. It can be seen from the figure that the violent crime offending rate begins to rise in the late1980s - just when the adolescents who should have been benefitting from the mandated reductions in average lead content of petrol from 2 g to 0.5 g per gallon in the 1975 to 1980 period were coming into the 12 to 17 age group. This uptrend continued to a peak in 1993 with generally high levels in the 1990-1995 years, even though all members of this age group in those years were born well after the mandated reductions began in 1975 to 1980. My point is not that lead plays no role in violent crime rates (almost surely, among children exposed to high levels of lead, the risk of crime offending will be higher than among children not so exposed) but that this is only one factor among many in determining overall societal crime rates and trends therein.

**Figure 1**: Trends in the violent crime offending rate among U.S. adolescents and teenagers ages 12-17 from the National Crime Victimization Surveys for the years 1975 to 2010 with projections for 2011 and 2012.

**Source: **Land, Kenneth C. 2013 NATIONAL Child and Youth Well-Being Index (CWI). New York: Foundation for Child Development.

I have read this submission. I believe that I have an appropriate level of expertise to confirm that it is of an acceptable scientific standard.

# Peer review: Referee response for version 2

**DOI:** 10.5256/f1000research.2664.r2248

**Published:** 2013-10-29

**Authors:** David Carpenter

**Affiliations:** 1 School of Public Health, University at Albany, NY, USA

My previous comments still apply. The one added sentence does not significantly change what I see to be a faulty conclusion of the data presented.

I have read this submission. I believe that I have an appropriate level of expertise to confirm that it is of an acceptable scientific standard, however I have significant reservations, as outlined above.

# Peer review: Referee response for version 1

**DOI:** 10.5256/f1000research.1877.r1933

**Published:** 2013-10-02

**Authors:** Kenneth Land

**Affiliations:** 1 Population Research Institute, Duke University, NC, USA

**Title and Abstract:** The title and abstract are appropriate to the content of the article.  The abstract represents a suitable summary of the work.

**Article content: **The article is a survey and review of prior research on the topic of whether or not the decline in all types of crime since the early 1990s in the USA was an unintended consequence of the elimination of lead from petrol between 1975 and 1985. This review is competently and adequately done for the most part. Its conceptual orientation comes from epidemiology, which is good, but the incorporation of the body of research done on this topic from a criminology perspective would add greater substance and depth to the article. The synthesis of these two perspectives would likely produce the inference that although the presence of lead in petrol was an influencing factor on the rise and subsequent decline of crime rates in the USA, it was but one of many factors and cannot account for these secular trends alone.

**Conclusions: **The conclusions are sensible, balanced and justified on the basis of the content of the survey and review of the existing research.

I have read this submission. I believe that I have an appropriate level of expertise to confirm that it is of an acceptable scientific standard.

# Peer review: Referee response for version 1

**DOI:** 10.5256/f1000research.1877.r1690

**Published:** 2013-09-05

**Authors:** Howard Mielke

**Affiliations:** 1 School of Medicine, Tulane University, LA, USA

This article is a good summary of the existing evidence on lead and violence and there's not much to dispute about it. However, a change in the perspective would improve it and at least one correction is needed.  

In the abstract the word ' *unintended*' is not correctly placed or in context of the problem. If anything, the unintended consequence is the plausible link between the toxic response due to rapid increase in the commercial consumption of fuel containing lead additives (especially in the 1950’s and 1960’s) and the unintended cascade of societal health costs, including learning disabilities and criminal behaviour, that was triggered around 20 years later. These consequences are only becoming appreciated recently, after the use of lead additives in fuels has decreased ^1^. 

Ecological studies like ours provide suggestive evidence, however when coupled with neuroimaging studies, animal studies and individual-level analyses, the current state of the science on lead provides strong evidence for a serious societal problem in need of attention to better protect future generations ^1^. The lead story is rich and so well-documented that the benefits of using resources for cleaning up play areas and improving the environment for children is becoming recognized by progressive nations as a cost effective and important activity ^2^.  

Correction: Page 5: “Meilke”--Mielke is misspelled.

I have read this submission. I believe that I have an appropriate level of expertise to confirm that it is of an acceptable scientific standard.

## References

[1] U.S. Department of Health and Human Sciences National Toxicology Program:: *Monograph on the Health Effects of Low-level Lead*.(2012); Reference source

[2] OttesenRTAlexanderJLangedalMHauglandTHøygaardE: Soil pollution in day-care centers and playgrounds in Norway: national action plan for mapping and remediation.*Environ Geochem Health*.2008;30: 156623-3710.1007/s10653-008-9181-x18563591

# Peer review: Referee response for version 1

**DOI:** 10.5256/f1000research.1877.r1532

**Published:** 2013-08-19

**Authors:** David Carpenter

**Affiliations:** 1 School of Public Health, University at Albany, NY, USA

This is a good review of the published literature, but the conclusions drawn by the author are not consistent with the data presented, specifically the statement “ *the evidence is not sufficient to conclude that variations in environmental lead exposure in childhood over the past 50 or so years in the USA explain, first the rise, and then the decline in crime rates*”. 

The evidence used to argue against this conclusion is primarily a study done in New Zealand, not in the US, plus a very limited cohort study by McCall and Land. 

The issue of concern is whether lead plays a role in rates of crime, and while one can argue about the percentage due to lead, the overall information certainly indicates that lead exposure is particularly important. This needs to be stated in a much more balanced fashion. Because this is the conclusion it is important that it be both balanced and supported by the data. If you discount lead exposure then there must be presentation of a credible alternative explanation, and none of the others are well supported by evidence.

I have read this submission. I believe that I have an appropriate level of expertise to confirm that it is of an acceptable scientific standard, however I have significant reservations, as outlined above.
